# Anti-Hypoxic Molecular Mechanisms of *Rhodiola crenulata* Extract in Zebrafish as Revealed by Metabonomics

**DOI:** 10.3389/fphar.2019.01356

**Published:** 2019-11-12

**Authors:** Yi Ma, Yi Wu, Zhengchao Xia, Jingyi Li, Xiaorong Li, Pingxiang Xu, Xuelin Zhou, Ming Xue

**Affiliations:** ^1^Department of Pharmacology, Beijing Laboratory for Biomedical Detection Technology and Instrument, School of Basic Medical Sciences, Capital Medical University, Beijing, China; ^2^Beijing Engineering Research Center for Nerve System Drugs, Capital Medical University, Beijing, China; ^3^Beijing Tropical Medicine Research Institute, Beijing Friendship Hospital, Capital Medical University, Beijing, China

**Keywords:** *Rhodiola crenulata*, anti-hypoxia, zebrafish, metabonomics, network analysis

## Abstract

The health supplement of *Rhodiola crenulata* (*RC*) is well known for its effective properties against hypoxia. However, the mechanisms of its anti-hypoxic action were still unclear. The objective of this work was to evaluate the molecular mechanisms of RC extract against hypoxia in a hypoxic zebrafish model through metabonomics and network pharmacology analysis. The hypoxic zebrafish model in the environment with low concentration (3%) of oxygen was constructed and used to explore the anti-hypoxic effects of RC extract, followed by detecting the changes of the metabolome in the brain through liquid chromatography–high resolution mass spectrometry. An *in silico* network for metabolite-protein interactions was further established to examine the potential mechanisms of RC extract, and the mRNA expression levels of the key nodes were validated by real-time quantitative PCR. As results, RC extract could keep zebrafish survive after 72-h hypoxia *via* improving lactate dehydrogenase, citrate synthase, and hypoxia-induced factor-1α in brains. One hundred and forty-two differential metabolites were screened in the metabonomics, and sphingolipid metabolism pathway was significantly regulated after RC treatment. The constructed protein-metabolites network indicated that the HIF-related signals were recovered, and the mRNA level of AMPK was elevated. In conclusion, RC extract had markedly anti-hypoxic effects in zebrafish *via* changing sphingolipid metabolism, HIF-related and AMPK signaling pathways.

## Introduction

Oxygen homeostasis is a survival prerequisite for most of the living creatures on the earth. Oxygen participates in the oxidation reaction for contributing necessary biology processing, and a suitable concentration of oxygen may drive carbon metabolism for energy production. When oxygen level reduces, some important pathways may be disturbed which could induce organ damage (Chaillou, 2018). Hypoxia, as a reduced oxygen environment, can induce the changes of physiology and pathology in the body. People existing at high altitude are damaged by hypoxia environment, and organ ischemia with low oxygen supply can also impair their health (Hiraga, 2018).

*Rhodiolae crenulatae* radix et rhizoma (“Hong-Jing-Tian” in Chinese; abbreviated as RC) is the rhizoma of *Rhodiola crenulata* (Hook. f. et Thoms.) H. Ohba which is widely grown in plateau and mountainous areas at high altitude. The health supplement and nutraceuticals of RC are widely used to treat hypoxia during hypoxia environment in Tibet of China (Li et al., 2015). People arriving at high altitude area from the plain have to take RC product for some days to conquer altitude stress, since there is no other effective medicine for anti-hypoxia except oxygen inhalation. RC also shows some potential pharmacological effects, such as anti-inflammation, neuroprotection, and anti-fatigue action (Lee et al., 2013; Chang et al., 2018). RC extract contains active components, such as salidroside, herbacetin, and quercetin, which are possibly responsible for anti-hypoxic activity (Gupta et al., 2010; Zheng et al., 2012). However, up to date, the anti-hypoxic mechanisms of RC are not fully explored.

Metabonomics obtains the profiles of endogenous metabolites to illustrate the significant disturbance of molecular and metabolism pathways (Laíns et al., 2018). After environmental stress or drug treatment, the metabolomes in the cell, tissue, organ, and/or body are changed. Compared to the genomics and proteomics, the metabonomic approach is an increasingly active tool to explore the key biological mechanisms which is most closely linking to phenotype. Herbal extracts have multiple active components for treating diseases *via* various targets. It’s unsuitable for herbs that only single protein target and pathway are explored for studying their mechanisms of action. Protein-protein interactions or protein-metabolite interactions are extensively used to investigate the pharmacological mechanisms of the herbs *via* multiple targets.

**Figure 1:**
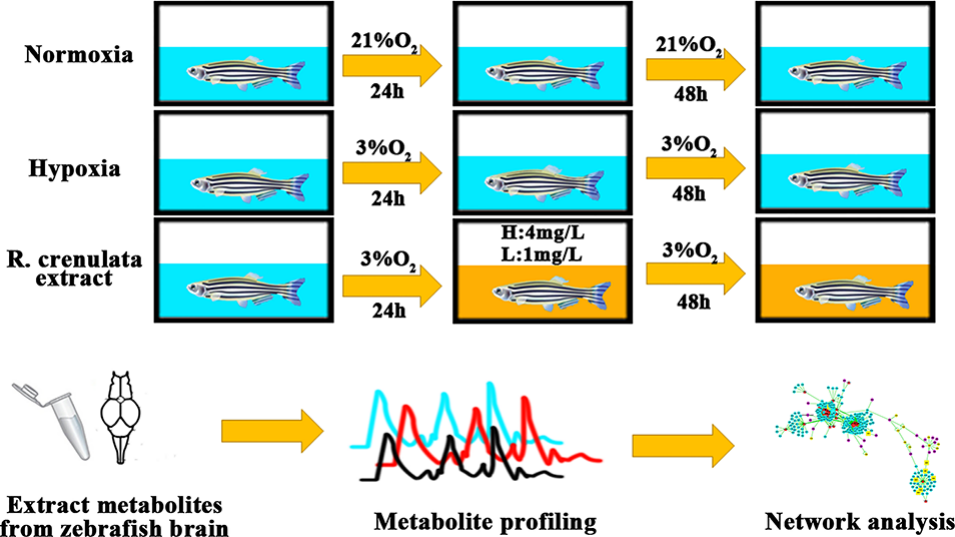
The process of zebrafish handling with different concentrations of oxygen. Background in blue indicates the normoxia or hypoxia environment and background in yellow shows RC extract treatment.

Zebrafish (*Danio rerio*) is a kind of model animals. Because of its short generation time, small size, and perfect fecundity, zebrafish model provides an excellent and wide application in pharmacological research (Jensen et al., 2011; Santos et al., 2017). Moreover, compared to other mammals, zebrafish model has a potential ability to adapt to hypoxia environment (Long et al., 2015). In recent researches, zebrafish embryos and larvae as animal models have been used to study hypoxia stress (Long et al., 2015). Thus, in our current study, we developed an adult zebrafish model under hypoxia to reveal the biological mechanisms of RC extract by using metabonomics and bioinformatics tools (**Figure 1**).

## Materials and Methods

### Chemicals and Materials

Acetonitrile (LC-MS grade), water (LC-MS grade), and formic acid (LC-MS grade) were purchased from the Fisher Scientific (Fair Lawn, NJ, USA). Methanol (HPLC grade) was obtained from the Fisher Scientific (Fair Lawn, NJ, USA). Power-up SYBGREEN was purchased from the Invitrogen Co. (Carlsbad, CA, USA). Authentic standard of salidroside (purity >98%) was purchased from the ChromaBio Co. (Chengdu, Sichuan, China). Authentic standards of rhodiosin, herbacetin, and kaempferol (purity >98%) were obtained from the YuanYe Co. (Shanghai, China).

### Plant Material and Herbal Extraction

The radix et rhizoma of *Rhodiola crenulata* (Hook. f. et Thoms.) H. Ohba was collected from the Xinjiang Region of China and supplied by the Kailai Biological Engineering Co., Ltd. (Xi’an, China). It was identified by Dr. Rong Luo (School of Chinese Medicine, Capital Medical University). A specimen (voucher number 2016-EJ819) was deposited in the Department of Materia Medica, School of Chinese Medicine, Capital Medical University.

The raw materials were powdered, and then extracted by heating reflux with 75% ethanol for two hours and repeated twice. After filtered, the combined solution was dried in vacuum for the collection of dry extract. The dried extract was stored in the desiccator before use. The yield of the dried extract was more than 20%.

### Chemical Composition Analysis

For the qualitation of the major components in the RC extract, Agilent 1100 HPLC-UV was used with a Grace Alltima C_18_ column (4.6 × 250 mm, 5µm). The mobile phase contained solution A (water with 0.2% formic acid) and solution B (acetonitrile). The gradient elution program was set as followed: 0–15 min, 5%–12% B; 15–40 min, 12%–15% B; 40–50 min, 15%–20% B; 50–80 min, 20%–25% B; 80–95 min, 25%–30% B; 95–110 min, 30%–35% B. The flow rate and detection wavelength were set at 1 ml/min and 280 nm, respectively. A typical HPLC-UV chromatogram of RC extract was shown in **Figure S1**.

For the quantitation of its major components, 1 mg of RC extract was weighed and dissolved in the solvent (methanol/water = 1:1) to the final volume of 1 ml. After sonicated for 30 min and centrifuged at 13,000 g for 10 min, the supernatant was collected. UPLC-MS/MS analysis was carried out using an Agilent 1290 system coupled with a triple-quadrupole Agilent 6490 mass spectrometer which was equipped with an ESI ionization source. The negative mode was used to quantify the amounts of active components. Chromatographic separation was performed by using the Waters Acquity HSS T3 column (2.1 × 150 mm, 1.8 µm). A gradient elution program separated all compounds with mobile phase A solution (0.1% formic acid in water) and B solution (0.1% formic acid in acetonitrile). The gradient elution program was performed as followed: 0–7 min, 10%–60% B; 7–8 min, 60%–100% B; 8–8.1 min, 100% B; 8.1–9 min, 100%–10% B; 9–10 min, 10% B. The total run time was 10 min. The ion pairs of standard active components and parameters of CE were listed as followed: salidroside, m/z 345.2→289.9, CE -10V; rhodiosin, m/z 609.1→301, CE -20V; herbacetin, m/z 300.9→255, CE -35V; kaempferol, m/z 285.0→92.8, CE -40V. The dwell time was 50 ms and fragmentor was 380V. The sample was tested in triplicate.

As shown in **Figure S1**, four components were identified with their respective authentic standards by LC-MS in the negative mode. The contents (w/w extract) of salidroside, rhodiosin, kaempferol, and herbacetin were 15.7 ± 0.1‰, 8.4 ± 0.1‰, 1.42 ± 0.06‰, and 0.95 ± 0.03‰, respectively.

### Zebrafish Handling

The wild-type TU zebrafish (mixed gender, 6 months old, 5 cm in length, 0.25 g in mass) were obtained from the Laboratory Animals Center of Capital Medical University (LACCMU, Beijing, China). Prior to the experiments, all zebrafish were acclimated under 14:10 h light-dark cycle and constant temperature (25 ± 2°C) for at least two weeks in a zebrafish feeding system (Far East Instrument Co., Ltd., Taiwan, China). The feeding system was equipped with 10-L tanks, a recirculating freshwater supplier, an air pump, a biological and mechanical filtration system, and ultraviolet lamps. The zebrafish husbandry and experiments were conducted by following the protocols approved by the Animal Experimentation Ethics Committee of Capital Medical University (AEEC No. AEEI-2016-016).

### Hypoxia Model and Drug Treatment

Adult zebrafish were placed in the chamber with pipes connecting with nitrogen gas flowing. After the environment has reached 3% of O_2_, the water chambers were pre-equilibrated with nitrogen gas for at least 2 h. The concentration of oxygen in the water and air was validated by an oxygen sensor (SX716, SANXIN, Shanghai, China). When nitrogen gas was balanced, the model group was exposed under hypoxia for 72 h. For RC treatment, the zebrafish were exposed to 24-h hypoxic treatment (3% oxygen), followed by administration of RC extract for 24-h and repeated administration for another 24 h. RC extract (lower than 100 mg/L) was totally dissolved in the water without observed precipitate. The LC_50_ value of RC extract in adult zebrafish was about 43 mg/L, and the two selected concentrations 4 mg/L and 1 mg/L of RC extract without observed toxicity were used for the above-mentioned treatment.

### Hypoxia-Related Biomarkers

After RC treatment, the zebrafish were sacrificed through putting into liquid nitrogen. The frozen brain samples were collected by forceps, weighed, and homogenized with cold phosphate buffered saline (PBS) solution. The activities of lactate dehydrogenase (LDH) and citrate synthase (CS) were tested by using the LDH and CS kit (Nanjing Jiancheng Co., China), respectively. HIF-1α was detected by the ELISA kit (Nanjing Jiancheng Co., China).

### Sample Preparation for Metabonomics

For metabonomic analysis, the frozen brain samples of another batch were weighed and homogenized with cold methanol and water (4:1, v/v) (Yuan et al., 2012). After incubated for 20 min and centrifuged at 13,000 rpm for 20 min at 4°C, the metabolites in brain were extracted in the supernatant. The samples were pooled as a quality control (QC) group. All samples were subjected to liquid chromatography–high resolution mass spectrometry (LC-HRMS).

### Metabonomics Analysis

LC-HRMS analyses were performed by using the Waters Acquity ultra-performance liquid chromatography (UPLC) coupled with Synapt G2-Si mass spectrometer which was equipped with data independent analysis (DIA) mode. The separation of polar metabolites was achieved through Waters Acquity HSS T3 (2.1 × 150 mm, 1.8 µm) column, while the nonpolar metabolites were separated by the Waters CSH C_18_ (2.1 × 150 mm, 1.7 µm) column. The parameters for mass spectrometry were listed as followed: capillary 3 kV for positive mode and 2.5kv for negative mode; sampling cone, 40V; source offset, 80V; cone gas, 50 L/h; desolvation gas flow, 600 L/h; source temperature, 120°C.

For the analysis of polar metabolites, the mobile phase program was set with 0.1% formic acid in water as mobile phase A and 0.1% formic acid in acetonitrile as mobile phase B. The gradient elution program for positive mode was performed as followed: 0–7.0 min, 0.1%–33% B; 7.0–12.0 min, 33%–44% B; 12.0–14.0 min, 44%–64% B; 14.0–18.0 min, 64%–100% B; 18.0–20.0 min, 100% B; 20.1–22.0 min, 0.1% B. Gradient elution program for negative mode was set as followed: 0–3.0 min, 0.1%–35% B; 3.0–6.0 min, 35%–44% B; 6.0–8.0 min, 44%–64% B; 8.0–13.0 min, 64%–100% B; 13.0–15.0 min, 100% B; 15.1–17.0 min, 0.1% B.

For the analysis of non-polar metabolites, the parameters were set with solution A (acetonitrile/water [60:40] containing 10 mM ammonium formate and 0.1% formic acid), and solution B (Isopropanol/acetonitrile [90:10] containing 10 mM ammonium formate and 0.1% formic acid). The gradient elution program for positive and negative modes was set as followed: 0–2.0 min, 40%–43% B; 2.0–2.1 min, 43%–50% B; 2.1–12.0 min, 50%–54% B; 12.0–12.1 min, 54%–70% B; 12.1–18.0 min, 70%–99% B; 18.1–20.0 min, 40% B.

The continuum data were collected from 50–1200 Da and the scan rate was 0.2 s. The representative chromatograms for metabonomics are presented in **Figure S2**. The QC samples were inserted to evaluate the stability of LC-MS system.

### Data Analysis

LC-MS data were analyzed using the Progenesis QI software (Nonlinear Dynamics, Newcastle, U.K.). After retention time and *m/z* were calculated, the peaks from each sample were aligned and picked. The isotope and adduct deconvolution were used to validate that the numbers of peaks were detected. Total ions were used for normalization. To obtain the metabolites from groups with significant differentials, the metabolites were scaled by the Pareto transformed. Ezinfo (3.0, Umetrics, Umea, Sweden) and SIMCA-P (13.0, Umetrics, Umea, Sweden) were used to achieve principle components analysis (PCA) and orthogonal partial least-squares discriminant analysis (OPLS-DA). The variable importance in the projection (VIP) value, *p*-value, and fold change from these metabolites were combined as the features to screen the differential metabolites. In addition, Benjamini and Hochberg method was used for the correction of false discovery rate (FDR). The compounds (VIP > 1, FDR < 0.05) were identified from Human Metabolome Database (HMDB), Kyoto Encyclopedia of Genes and Genomes (KEGG), and LipidMaps databases. Pathway analysis and enrichment for metabolites were analyzed by Metaboanalyst 4.0 (www.metaboanalyst.ca) (Chong et al., 2018). The QC samples were conducted for PCA score to assess LC-MS stability.

### Interaction Network Construction Between Protein and Metabolites

To reveal the anti-hypoxic mechanisms of action of RC extract, MBROLE v.2.0 (http://csbg.cnb.csic.es/mbrole) was used to construct potential protein-metabolite interaction and enrichment analysis (López-Ibáñez et al., 2016). The likely proteins of hypoxia interacting with metabolites were collected for further analysis using the OMIM, HDMB, and STRING protein-protein interaction databases. The visualization of the formed network was achieved by Cytoscape v.3.6. In order to realize the function of a disturbing protein in the hypoxia group or treatment group, the clusters were applied to show protein functions.

### Validation of Potential Targets

Total RNA from the brain was extracted using Trizol (Sigma-Aldrich, USA). RNA was reversely transcripted to cDNA by ReverTra Ace (TOYOBO, Japan) *via* the Oligo dT primers. PCR SYBR master mix (Invitrogen, USA) was used for amplification of each potential target. The cycling conditions were listed as followed: 50°C, 2 min for activation; 95°C, 2 min for DNA polymerase; 95°C, 15 s for denaturing; and 60°C, 1 min for annealing. Processes of denaturing and extending were repeated for 40 times. The sequences of primers are available in **Table S1**. Relative mRNA expression was calculated through normalization with the housekeeping gene *gapdh*.

## Results

### Hypoxia Biomarkers

**Figure 2:**
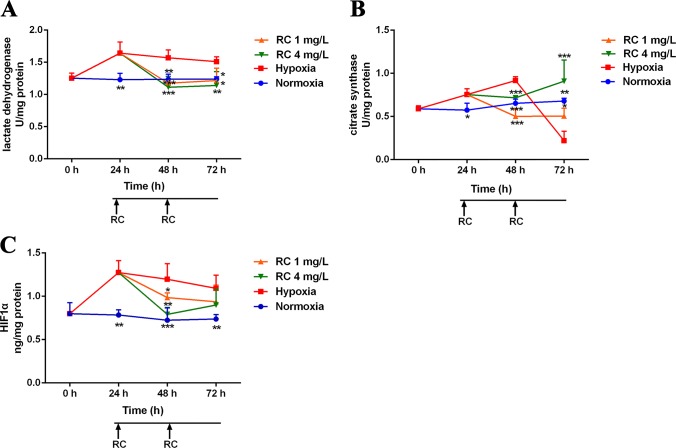
The activities of hypoxic biomarkers including **(A)** lactate dehydrogenase, **(B)** citrate synthase, and **(C)** hypoxia-induced factor-1α in different RC extract treatment groups under hypoxia when compared to the hypoxia and the normoxia groups. *p < 0.05, **p < 0.01, and ***p < 0.001 versus the hypoxia control group. Data are presented as the mean ± SD (n = 4).

RC extract could markedly improve the survival of zebrafish in the hypoxic environment (3% oxygen). Meanwhile, the activities of LDH and HIF-1α were significantly declined in the RC group, when compared to those in the hypoxia group (**Figure 2**). The activity of CS was rapidly reduced which may be due to the injury in the hypoxic environment. However, the levels of LDH, HIF-1α, and CS in the normoxia group were stable during the measurements. In the RC treatment group under normoxia, as illustrated in **Figure S2**, the hypoxic biomarkers showed no significant change. Thus, RC extract could keep homeostasis for the hypoxia-related biomarkers, indicating that RC had excellent anti-hypoxia effect under hypoxic environment.

### Multivariate Data Analysis in Metabonomics

**Figure 3:**
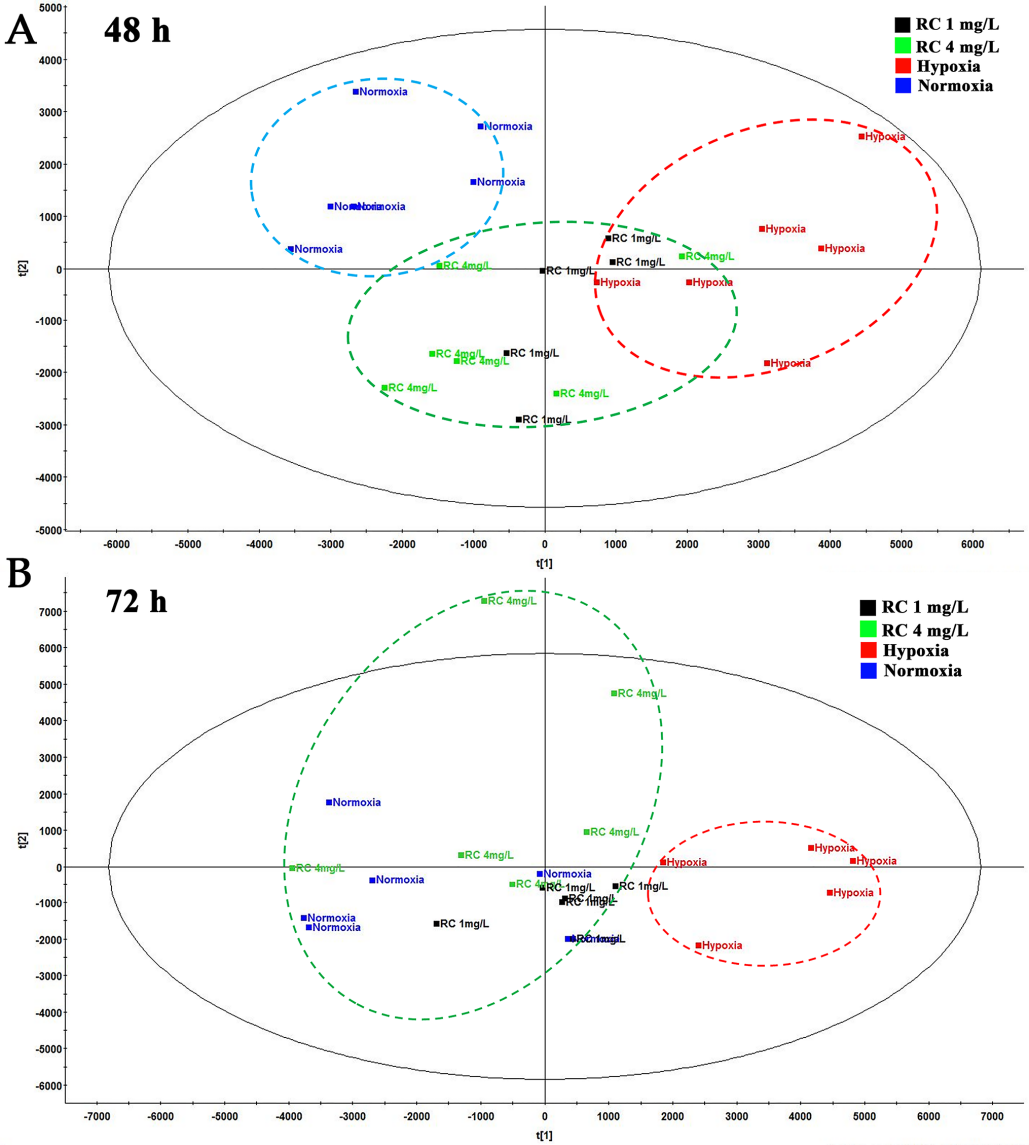
The PCA plot among normal, hypoxia, and RC extract treatment groups. The red dash circle shows hypoxia control. Blue dash circle shows normoxia control. **(A)** The PCA score among normal, hypoxia and treatment after 48-h treatment. **(B)** The PCA score among three groups after 72-h treatment (n = 5).

To evaluate the anti-hypoxic mechanisms of RC extract, the metabolites in different groups were separated and analyzed by the positive and negative modes of base peak intensity chromatograms which were obtained from LC-HRMS. The profiles of base peak intensity were illustrated in **Figure S3**. The closely gathered plots of the QC group showed good stability of the LC-MS system, as illustrated in **Figure S4**. After normalization and peaks alignment *via* the QI software, these peaks were picked from the different groups to construct multivariate analysis in EZinfo and SIMCA-P, followed by multivariate statistical approaches of PCA. The PCA plots (R^2^ = 0.79, Q^2^ = 0.50) clearly separated normoxia and hypoxia group in the metabolic state, and the changes of metabolome were in a time-dependent manner, as depicted in **Figure S5A**. After RC extract treatment, the metabolite traces became similar to the normal group. As depicted in **Figure S5B**, the different metabolites were recovered after RC treatment as the PCA plots illustrated. Moreover, the plots of 24-h treatments of 4 mg/L and 1 mg/L of RC were similar to that of hypoxia group in **Figure 3A**, whereas the plots of 72-h RC treatment at 4 mg/L were almost similar with that of normoxia group in **Figure 3B**. The results indicated that RC had a better effect at higher concentration (4 mg/mL).

### Pathway Analysis

**Figure 4:**
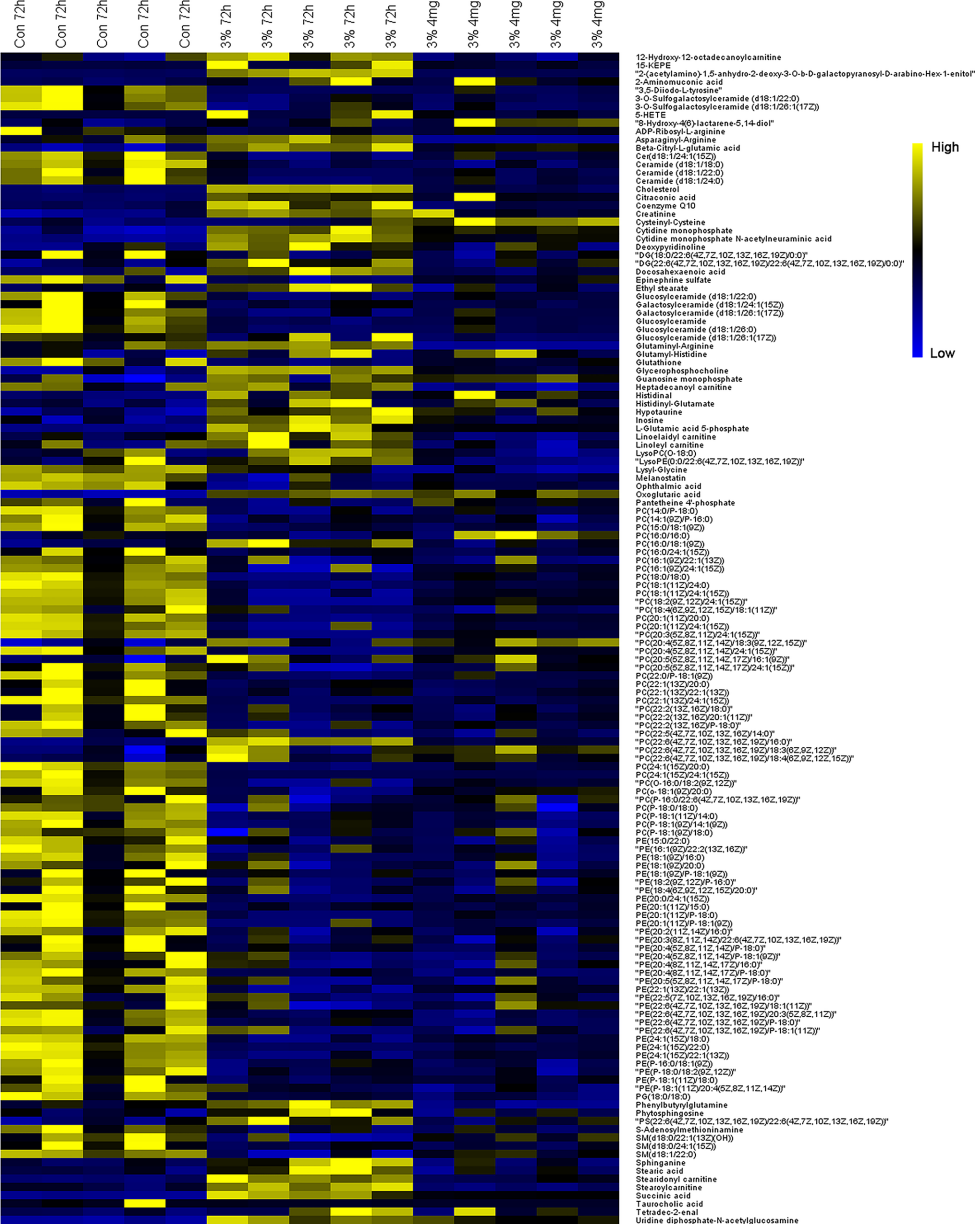
The heatmap of metabolites among normoxia, 72-h hypoxia and RC extract (4 mg/L) treatment. Metabolites labeled in yellow or blue indicate the high or low levels, respectively.

**Figure 5:**
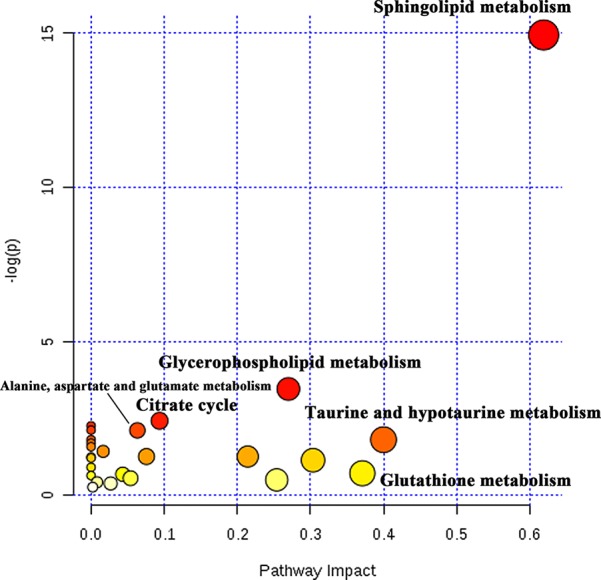
The pathway analysis of differential metabolites. The tendency of red circles shows the importance of metabolism pathway.

Potential biomarkers with VIP value more than 1 and FDR value lower than 0.05 were selected from loading plots *via* OPLS-DA and ANOVA analysis. The metabolites with fold change over 1.2 were collected. Several databases (e.g. HMDB, KEGG, and LipidMaps) were used to identify these compounds *via* fragment peaks by data-independent acquisition mode. One hundred and forty-two of the total metabolites were identified when compared with the metabolomes among the normoxia, hypoxia, and 4 mg/L of RC groups, and their chemical information was shown in **Table S2**. Besides, as depicted in **Figure 4**, the relative concentration trends of numerous differential metabolites were recovered by RC treatment. The enrichment and pathway analysis showed that the pathways of sphingolipid metabolism, glycerophospholipid metabolism, and citrate cycle were obviously disturbed under hypoxia and re-adjusted after RC extract treatment (**Figure 5**). The taurine and hypotaurine metabolism, as well as alanine, aspartate, and glutamate metabolism, were also changed after RC extract treatment under hypoxia.

### Network Construction of Protein and Metabolites Interaction

**Figure 6:**
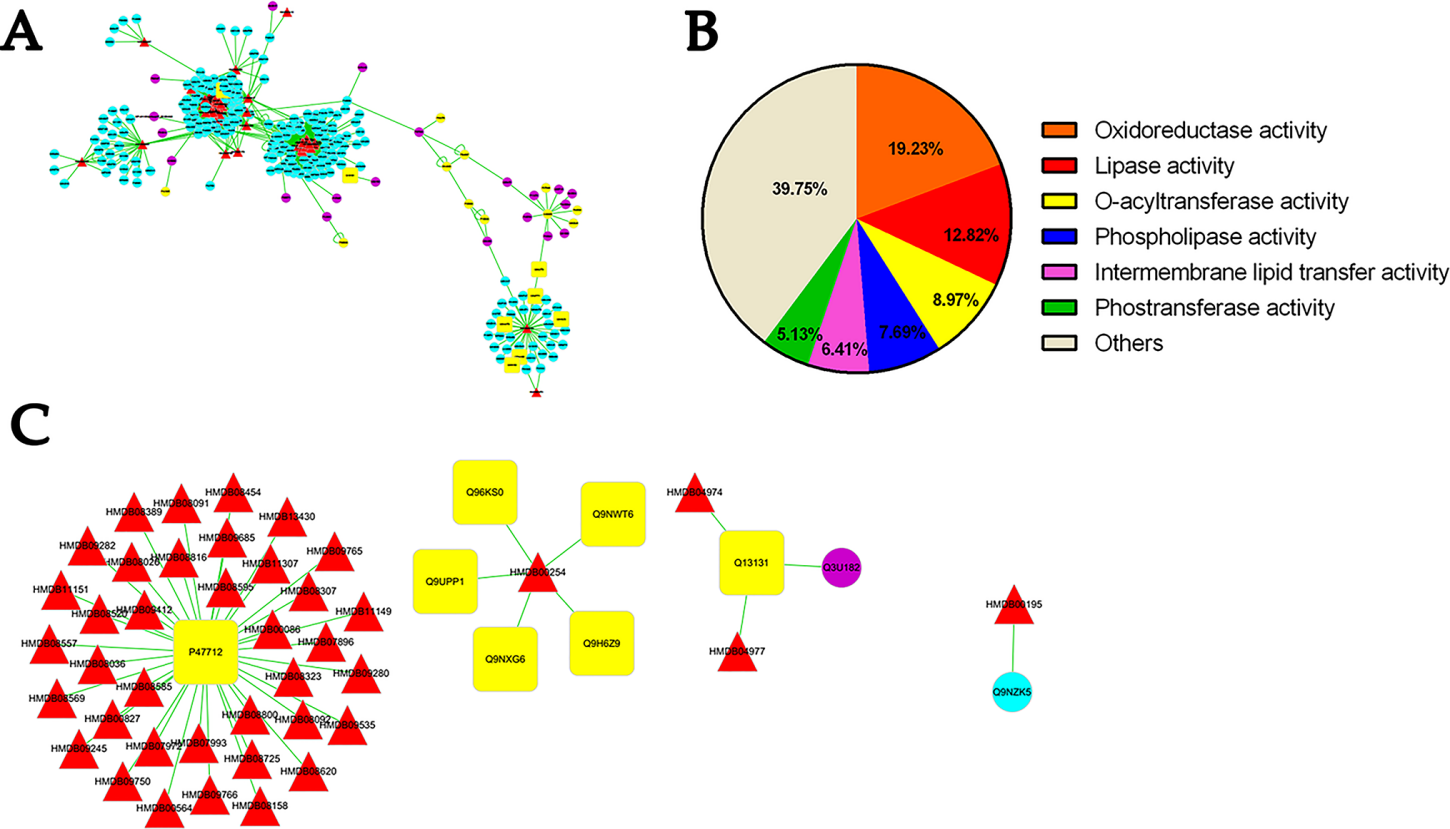
The analysis of protein-metabolite interaction network. Whole network **(A)**, the molecular functions of the selected proteins **(B)**, and the first neighbor node of proteins linking to metabolites **(C)** indicate the interactions between protein and metabolites. Red triangle shows differential metabolites *via* metabonomics and blue circle shows their interacting proteins listed in the MBRole database. Yellow circle indicated interacting proteins with metabolites straightly correlated to hypoxia. Q9UPP1: PHF8; Q9NWT6: HIF1α; Q9GZT9: EGLN1; Q96KS0: EGLN2; Q9H6Z9: EGLN3; Q9NXG6: P4HTM; Q13131: PRKAA1; P47712: PLA2G4A.

The cluster analyses were established to reveal several functions influenced by RC treatment as depicted in **Figure 6**. The most influenced part is oxidoreductase and lipase activity. Moreover, O-acyltransferase and phospholipase may also participate in the anti-hypoxic function of RC extract. The metabolite-protein-hypoxia interaction network was built to explore the hub nodes of RC extract treatment. In order to directly obtain interaction with the metabolites in hypoxia, the first neighbor nodes of interaction network were conducted. As associated with RC treatment under hypoxia, there were some key proteins including histone lysine demethylase (PHF8), hypoxia-inducible factor 1-alpha (HIF-1α), Egl nine homolog 1 (EGLN1), Egl nine homolog 2 (EGLN2), Egl nine homolog 3 (EGLN3), transmembrane prolyl 4-hydroxylase (P4HTM), 5’-AMP-activated protein kinase catalytic subunit alpha-1 (PRKAA1), and cytosolic phospholipase A2 (PLA2G4A).

**Figure 7:**
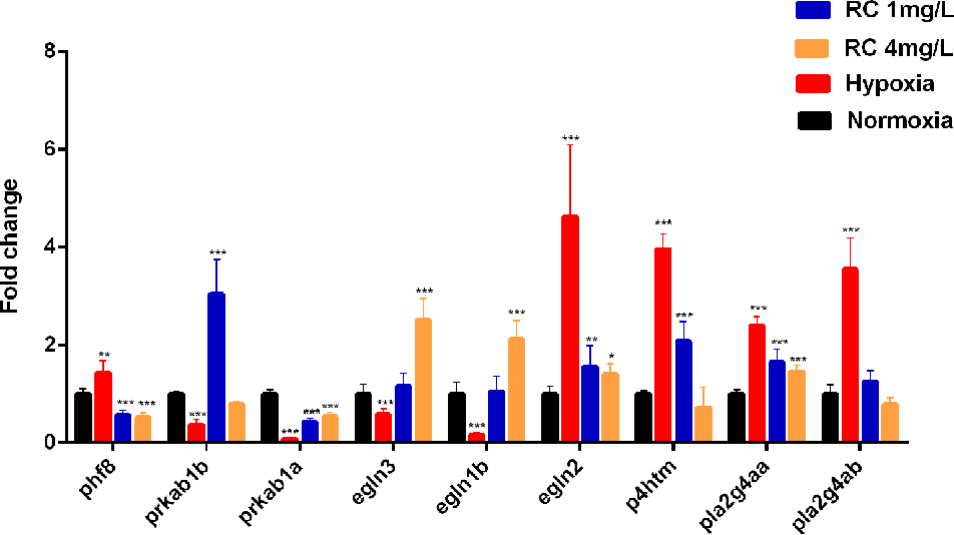
The mRNA expression of key node in zebrafish brain tissue after different RC extract treatment under hypoxia, as detected by real-time PCR. *p < 0.05, **p < 0.01, ***p < 0.001 versus hypoxia groups. Data are presented as mean ± SD (n = 6).

To verify this network, the mRNA levels were quantified by real-time PCR. The results showed that these genes obviously changed in different treatment groups (**Figure 7**). The encoded genes of HIF prolyl-hydroxylase (PHD) including *egln1b* and *egln3* were elevated under hypoxia, whereas their expressions were decreased after RC treatment, while *egln2* had a reverse tendency. As shown in the protein-metabolite network, *p4htm* was connected with succinic acid, and its mRNA level was decreased by RC treatment. As encoding AMPK kinase, the mRNA expression levels of *prkab1b* and *prkab1a* were improved in RC treatment group when compared to hypoxia group. Moreover, the mRNA level of *phf8* was decreased *via* RC treatment when compared to the hypoxia group. As important hub nodes interacted with the multiple differential metabolites including phosphatidylcholine and phosphatidylethanolamine, the mRNA levels of *pla2g4aa* and *pla2g4ab* were disturbed under hypoxia, and reversed after RC treatment.

## Discussion

*Rhodiola crenulata* is a well-known traditional Chinese medicine (TCM) reported to enhance hypoxia endurance capacity. RC extract was widely used to treat high altitude illness and mountain sickness (Chen et al., 2014). Our results supported the anti-hypoxic effects of RC in hypoxic environments, and RC could maintain zebrafish survival, which are consistent to its clinical effect.

LDH, HIF-1α, and CS possess different functions in the hypoxic environment. LDH, reflecting the degree of hypoxia, is a potential biomarker for hypoxic injury in clinical diagnosis (Fineschi et al., 2017). HIF-1α is a sensitive oxygen sensor that has different biological functions in hypoxia. Our results indicated that RC extract could decrease LDH activities and HIF-1α expression which were elevated under hypoxia. The activity of citrate synthase, a crucial enzyme to produce citrate, indicates the capacity of aerobic respiration and mitochondrial function (Larsen et al., 2012). Mikaela et al. showed that high altitude and chronic hypoxia could increase CS activities (Mandic et al., 2013; Lui et al., 2015). Similar to our data, the CS activity in hypoxia group increased after 48-h hypoxia condition, indicating that the zebrafish enhanced the CS activity to conquer hypoxia in the first 48-h hypoxic treatment. However, if mitochondria were injured, CS would decrease in hypoxic environment (Warren et al., 2017). This is similar to our results on CS activity which was rapidly declined after 72-h serious hypoxia, showing that after 72-h hypoxic treatment, the mitochondria of zebrafish possibly were damaged. Meanwhile, to resemble with the tendency of normoxia group, CS activity was kept to be steady by RC extract treatment. In brief, our results indicated that RC treatment improved these direct hypoxia-related indicators, suggesting that RC could keep zebrafish adaptation in hypoxia environments.

The PCA scores in metabonomics illustrated that the plots of higher concentration of RC extract were closed to the plot of normoxia group, meaning that RC had an excellent effect on anti-hypoxia through regulating endogenous metabolites. Because TCM contains multiple active components, the network pharmacology provides an appropriate approach, as a tool for system biology, to fully understand the mechanisms of actions (Zhang et al., 2019). For RC extract, the multiple molecular targets could be influenced by RC treatment to improve the anti-hypoxic ability of organs. In our study, the pathway analysis and metabolite-protein interaction network were conducted, indicating that sphingolipid metabolism, HIF-1α related and AMPK signaling pathways were obviously regulated under hypoxia.

Sphingolipid metabolism is implicated in various signaling pathways and pathological changes such as cell membrane injury and inflammation. Sphingomyelin (SM), an important molecule in sphingolipid metabolic pathway, is essential for the formation of plasma membrane and involves in cell proliferation and growth (Adada et al., 2016; Bienias et al., 2016). So far, several studies have proved that the elevated concentration of SM plays a vital role in the stabilization of cell membrane against hypoxia injury (Zager, 2000; Moschetta et al., 2000). LDH activity is a common biomarker to reflect cell injury and cell integrity, and it decreased *via* RC treatment. In addition, low concentration of oxygen could reduce the SM level, however, RC could recover its level. Thus, as change of sphingolipid metabolic pathway after RC treatment, the increased level of SM was consisted with the decrease of LDH activity, indicating that RC treatment might enhance cell protection to resist hypoxia. Meanwhile, the activities of lipase and phospholipase were also altered obviously with regulation of sphingolipids. Several researches have provided that sphingolipid metabolism influences not only lipase activity, but also the activity of cytosolic phospholipase A2 (cPLA2) (Nakamura et al., 2010; Nakamura and Murayama, 2014). The cPLA2 increases the level of arachidonic acid, leading to inflammatory reactions by disrupting endogenous metabolic balance (Meirer et al., 2014). Two genes *pla2g4aa* and *pla2g4ab*, encoding cPLA2 in zebrafish, were up-regulated in hypoxic environment, but recovered after RC treatment. In addition, two metabolites, 5-hydroxyeicosatetraenoic acid (5-HETE) and 15-Oxo-ETE (15-KETE), were enriched in arachidonic acid metabolic pathway, which were inflammatory factors (Samuel et al., 1993; Powell and Rokach, 2015). With declining of cPLA2, they were suppressed *via* RC treatment, indicating that inflammation was alleviated. In summary, RC treatment could increase cellular sphingomyelin as a critical molecule in sphingolipid metabolism, and finally cause the decline of cPLA2, an inflammatory producer.

HIF-1α is a well known and hub biomarker for hypoxia. Our results indicated that the level of HIF-1α decreased after RC treatment, which was consistent to the data of the mRNA expression levels of genes regulating HIF-1α. PHF8 plays an essential role in hypoxia stress, and its knockdown can reduce the activation of HIF-1α (Maina et al., 2017). EGLN1, EGLN2, and EGLN3 are a family of proteins which can influence hypoxia process (Schofield and Ratcliffe, 2004; Ivan and Kaelin, 2017). The *egln1*, as called *egln1b* in zebrafish, encodes PHD2 to degrade HIF-1α by prolyl hydroxylation in an aerobic environment (Schofield and Ratcliffe, 2004). The mRNA expression of *egln1b* in treatment group was elevated when compared to hypoxic group. PHD1 and PHD3 were also encoded by *egln2* and *egln3*, respectively. Similar to the expression tendency of *egln1b*, the mRNA level of *egln3* was reduced under hypoxia but increased after RC treatment. However, *egln2* had an opposite trend in an aerobic environment when compared to hypoxia. It is possible that PHD2 is main regulator and PHD3 is most relevant isoform for HIF-1α (Appelhoff et al., 2004). P4HTM also plays essential roles in the HIF-related signaling pathway to balance oxygen homeostasis, and inhibition of P4HTM is regarded as a potential target for treating ischemia (Hyvärinen et al., 2010). Our work showed that the mRNA expression of *p4htm* was almost four-fold changes under hypoxia whereas its expression level in RC (4 mg/L) group were similar to that in the normoxia group.

Alternatively, citrate cycle has a critical role in essential energy metabolism. Succinate, as a key metabolic intermediate in TCA circle (Tretter et al., 2016; Lukyanova et al., 2018), regulates the transcription of HIF-1α both in the Krebs cycle and in GABA shuttle. After RC treatment, the treatment group declined the concentration of succinate so as to increase HIF-1α degradation, which was consistent to the above-mentioned ELISA results. By elevating HIF-1α signal pathway, some researchers have revealed that it could increase inflammatory cytokines to up-regulate the formation of arachidonic acids which are inflammatory factors (Pchejetski et al., 2010; Tannahill et al., 2013; Guo et al., 2017). Thus, the declined HIF-1α could cause down-regulation of arachidonic acid, which partly may reduce the inflammatory effect and subsequently alleviate hypoxia damage.

In addition, *in silico* network construction indicated that AMP-related signaling pathway was also elevated. AMPK involved in regulating energy metabolism in hypoxic environment. As an AMP-activated protein kinase and an analog with PRKAB1B and PRKAB1A in zebrafish, PRKAA1 can improve hypoxia tolerance under nutrient-deprived conditions (Sun et al., 2018). Lee et al. showed that RC extract has potential to exert the glucose-lowering effect *via* activating AMPK signaling pathway (Lee et al., 2015). This evidence was consistent with our results, and supported that AMPK signaling pathway played a crucial role in the regulation of RC extract against hypoxia. During hypoxia for three days, *prkab1a* and *prkab1b* were inhibited. It’s likely exhausted in the hypoxic environment due to the acceleration of energy consumption. In contrast, the expressions of these genes were increased after RC treatment, suggesting that these genes had actions on energy homeostasis. After 72-h hypoxia treatment, CS activity was obviously suppressed in the hypoxic group which may be due to the inhibition of AMPK and the exhaustion of energy, whereas RC treatment could keep energy homeostasis.

Nevertheless, we confirmed some important metabolites based on the databases. A further study should be performed for the exact mechanisms of RC extract to anti-hypoxia action *via* the molecular and systemic biology approaches.

## Conclusion

**Figure 8:**
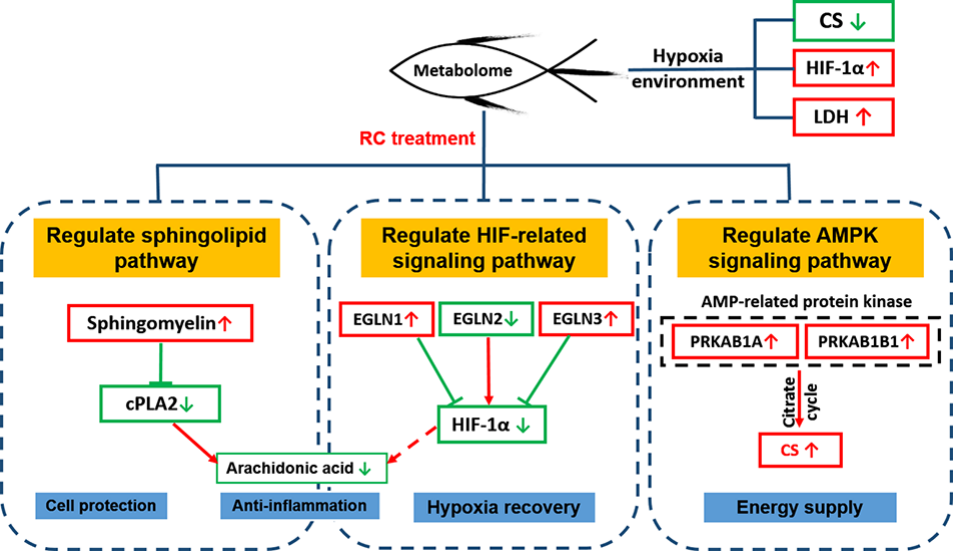
The molecular mechanisms of RC extract against hypoxia in zebrafish revealed by metabonomics and *in silico* network

Taken together, our work firstly used metabonomics and hypoxic zebrafish model to reveal the metabolic pathway recovered by RC extract. Differential metabolites based on the databases were screened and the interaction network between the protein and metabolites, as regulated by RC treatment, was constructed. As depicted in **Figure 8**, RC shows an excellent effect on anti-hypoxia *via* multiple molecular mechanisms. Sphingolipid metabolism, HIF-related and AMPK signaling pathways were regulated by RC extract to keep zebrafish survive under hypoxia. Our investigations provided not only an insight that zebrafish could be a tool to study hypoxia, but also a systematic understanding of anti-hypoxic molecular mechanisms of RC extract.

## Data Availability Statement

All datasets generated for this study are included in the article/**Supplementary Material**.

## Ethics Statement

The animal study was reviewed and approved by Animal Experimentation Ethics Committee of Capital Medical University.

## Author Contributions

YM and MX designed the study and wrote the article. YW, ZX, and JL carried out experiments. XL and PX developed analysis tools. XZ and MX gave advice and careful revision.

## Conflict of Interest

The authors declare that the research was conducted in the absence of any commercial or financial relationships that could be construed as a potential conflict of interest.

## Abbreviations

RC, *Rhodiola crenulata*; LDH, citrate synthase; HIF-1α, hypoxia-induced factor-1α.
