# Supplementary material for: Anti-Hypoxic Molecular Mechanisms of Rhodiola crenulata Extract in Zebrafish as Revealed by Metabonomics
Source: Front Pharmacol. 2019 Nov 12;10:1356. doi: 10.3389/fphar.2019.01356 (PMC6861209; doi:10.3389/fphar.2019.01356)
Supplement: Supplementary file 1 [file DataSheet_1.docx]

**
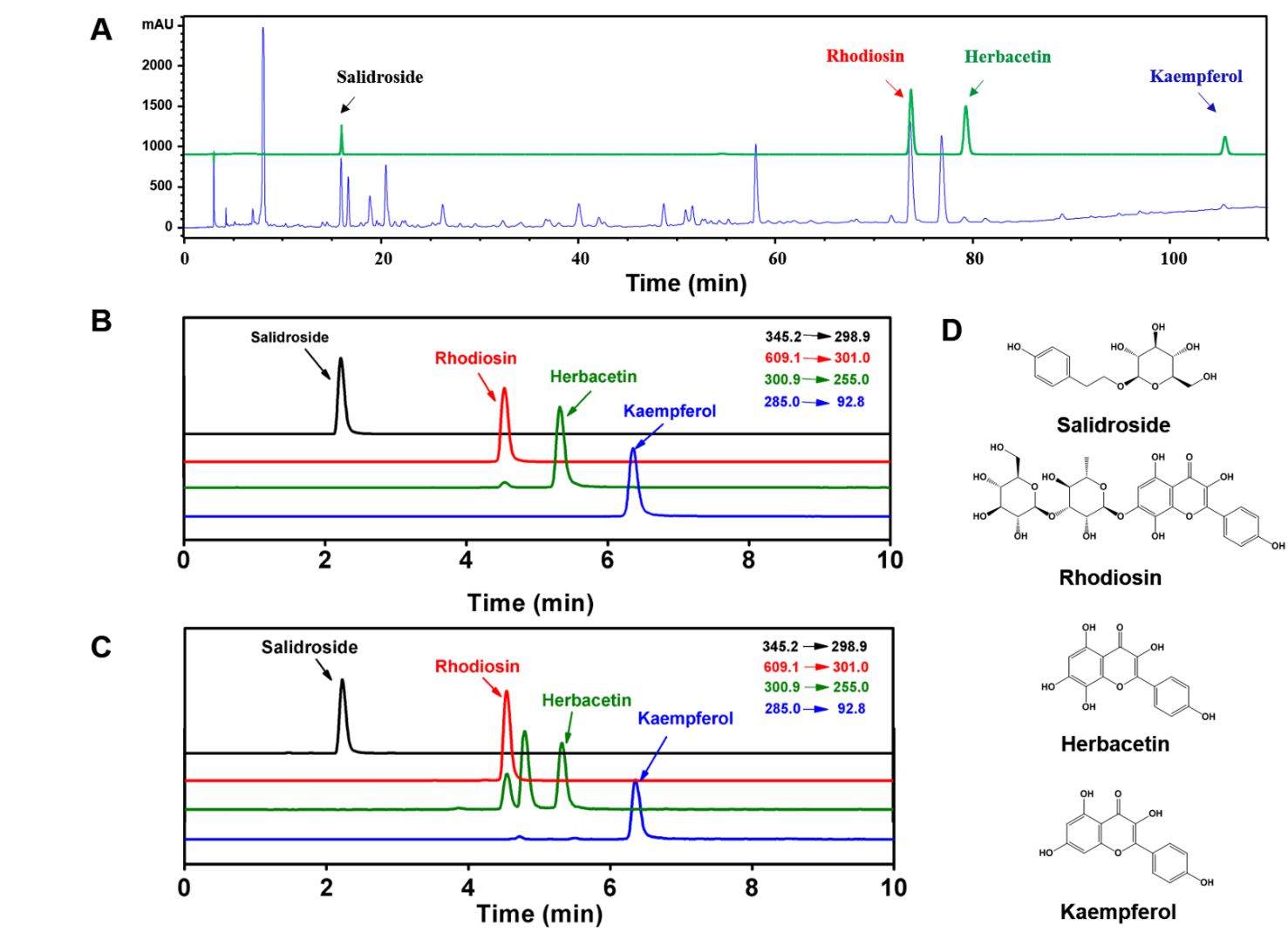
**

**Figure S1.**Chemical profile and composition analysis of RC extract. (A) HPLC-UV chromatograms (at 280nm) of RC extract (in blue) and four standards (in green); Extracted ion chromatograms of four standards (B) and RC extract (C) as detected by negative MRM mode. (D) The chemical structures of four chemical standards (Purity > 98%) including salidroside, rhodiosin, herbacetin and kaempferol.

**A B**

**



**

**C**

**

**

**Figure S2.** The activities of hypoxia biomarkers including (A) lactate dehydrogenase, (B) citrate synthase and (C) hypoxia-induced factors were not significantly changed after administration of RC extract (1mg/L or 4mg/L) under normoxia. Data are presented as mean ± SD (n=4).

**

A**

**

B**

**Figure S3**. The representative base intensity chromatograms (BPC) of metabolite profiling of zebrafish brain homogenate. (A) BPC in red shows metabolites separated by HSS T3 column; (B) BPC in blue shows lipid metabolites separated by CSH C_18_ column.


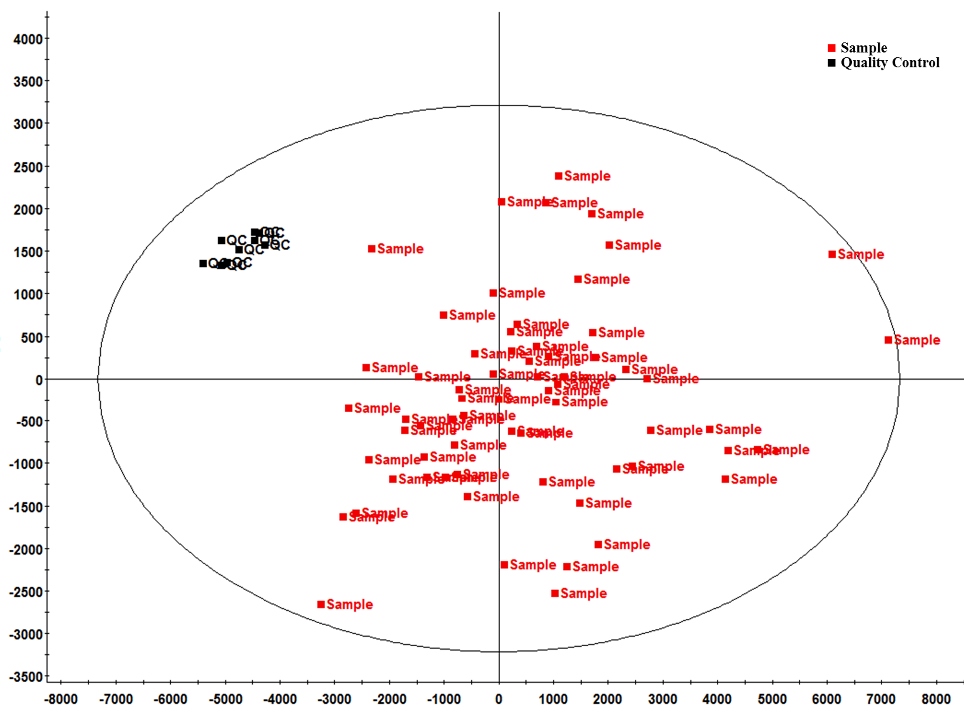


**Figure S4.** The Quality controls (QC) and samples are illustrated in PCA scores. The black labels are for QC group and the red ones are for the samples.

**A
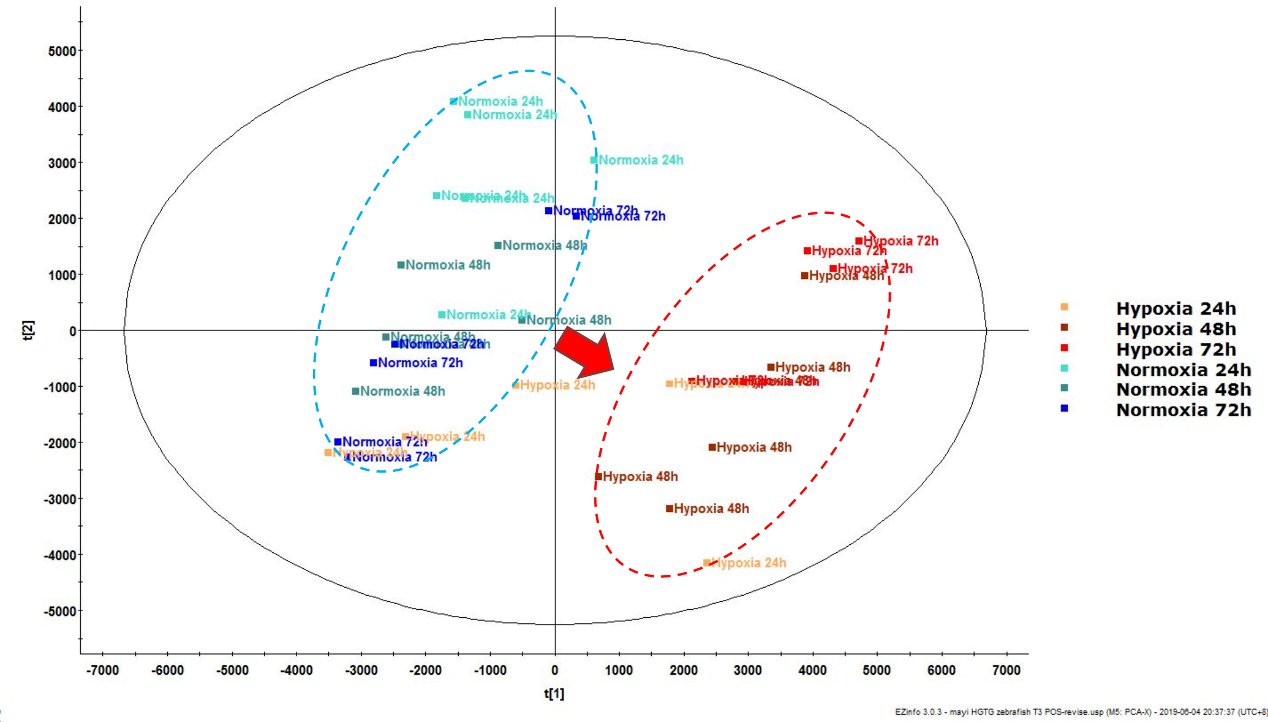
**

**B**

**
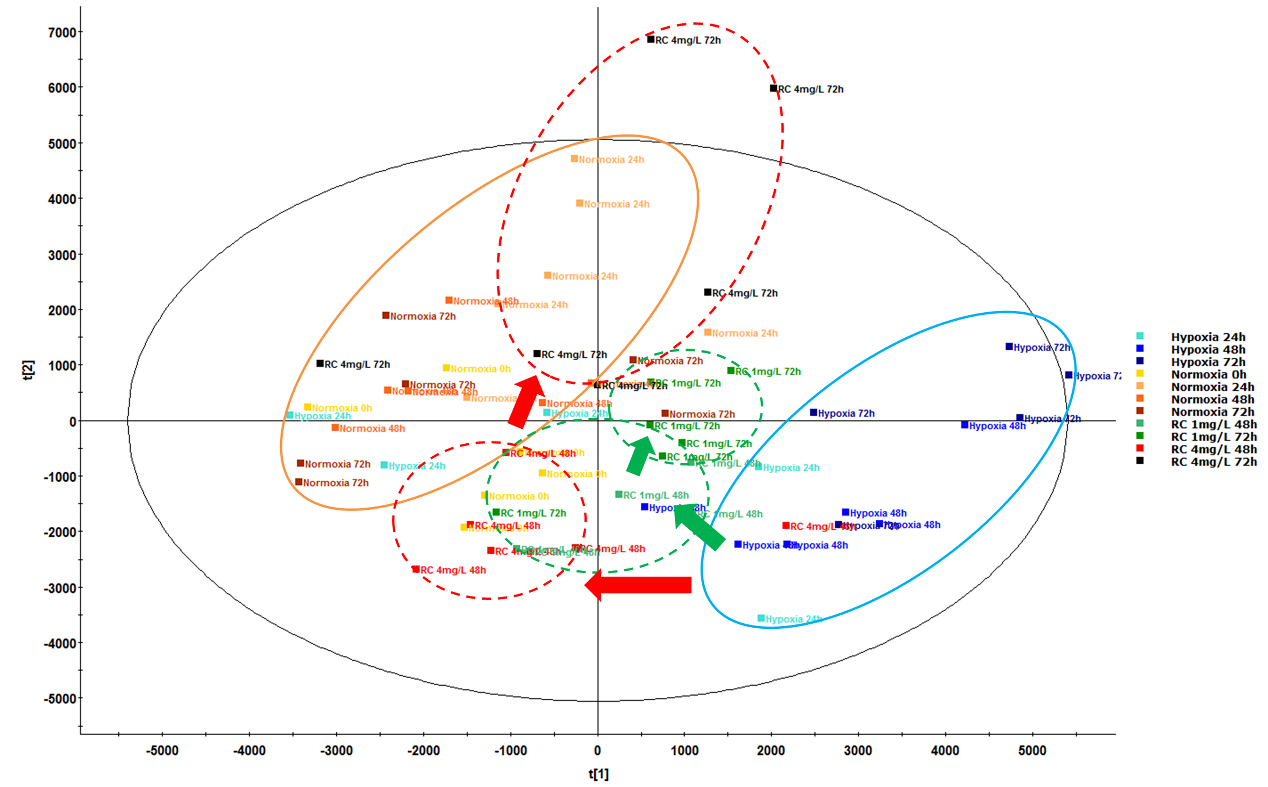
**

**Figure S5.** The metabolome traces in different groups. (A) The time-dependent trace in hypoxia and normoxia group from 24 to 72h. The blue plot is for normoxia group and the red one is for hypoxia group. (B) The PCA score among normal, hypoxia and different treatment groups of RC extract. The blue circle shows hypoxia control. Red or green dash circles show the time trends of metabolites after RC treatment at high dose (4 mg/L) or low dose (1 mg/L), respectively. (n=5).

**Table S1** The information for PCR primers. (F, forward; R, reward).

| Gene | Encoding protein | Primers (5-3) |
| --- | --- | --- |
| gapdh | Glyceraldehyde-3-phosphate dehydrogenase | F: TTCCAGTACGACTCCACCCA |
|  |  | R: TGACTCTCTTTGCACCACCC |
| egln1b | Egl nine homolog 1 | F: GCGGGGATAAGATCACCTGG |
|  |  | R: CAAAAGGCCACCATGTTCCG |
| phf8 | Histone lysine demethylase | F: AAACGGCGAGGAGCTGTAAA |
|  |  | R: GGACTGGGACACTGAACGAG |
| egln2 | Egl nine homolog 2 | F: TAAGAACATTCGCGGGGACC |
|  |  | R: CGCCCGTCACCATTAGGATT |
| p4htm | Prolyl4-hydroxylase, transmembrane | F: GTCAGGCAAAGTCCACGAGA |
|  |  | R: TGGCCATCTTGGTTGAGGTC |
| prkab1a | Protein kinase, AMP-activated, beta 1 non-catalytic subunit, a | F: CAGGCCCATACCATCAGGAC |
|  |  | R: CTCTGGGAGCAAAGCTGGAT |
| prkab1b | Protein kinase, AMP-activated, beta 1 non-catalytic subunit, b | F: AACTGCGCTCTCCACCTATC |
|  |  | R: GGTGGTTGAGCATCACATGG |
| egln3 | Egl-9 family hypoxia-inducible factor 3 | F: CCTGTGGGCTTCTCAACGA |
|  |  | R: TTTGACCTCGCACGAATGCT |
| pla2g4aa | Phospholipase A2 | F: ACCTGCCCTACCCTCTCATT  R: AACTCTTTGAACGGTGGGCT |
| pla2g4ab | Phospholipase A2 | F: CCAACCAAGACAACGTGCTG  R: GAATCGCAGGTCCATCGCTA |
